# Anti-inflammatory protein TNFα-stimulated gene-6 (TSG-6) reduces inflammatory response after brain injury in mice

**DOI:** 10.1186/s12865-021-00443-7

**Published:** 2021-08-04

**Authors:** Kazadi Nadine Mutoji, Mingxia Sun, Amanda Nash, Sudan Puri, Vincent Hascall, Vivien J. Coulson-Thomas

**Affiliations:** 1 College of Optometry, University of Houston, 4901 Calhoun Road, Houston, TX 77204-2020 USA; 2 Cleveland Clinic, Cleveland, OH USA; 3 Present Address: Department of Bioengineering, Rice University, Houston, TX 77030 USA

**Keywords:** TSG-6, Glial scar, Astrocytes, Glycosaminoglycans and inflammation

## Abstract

**Background:**

Current research suggests that the glial scar surrounding penetrating brain injuries is instrumental in preserving the surrounding uninjured tissue by limiting the inflammatory response to the injury site. We recently showed that tumor necrosis factor (TNF)-stimulated gene-6 (TSG-6), a well-established anti-inflammatory molecule, is present within the glial scar. In the present study we investigated the role of TSG-6 within the glial scar using TSG-6 *null* and littermate control mice subjected to penetrating brain injuries.

**Results:**

Our findings show that mice lacking TSG-6 present a more severe inflammatory response after injury, which was correlated with an enlarged area of astrogliosis beyond the injury site.

**Conclusion:**

Our data provides evidence that TSG-6 has an anti-inflammatory role within the glial scar.

## Background

Traumatic brain injury (TBI) is a major medical concern that affects over 10 million people in the world each year [1, 2]. A variety of injuries can cause TBI leading to a range of injury severities [3–7]. With improved medical interventions over the years, the mortality rate due to TBI has decreased, resulting in a significant number of people living with the long-term effects of TBI. It is well accepted that in addition to the immediate effects of TBI there are also multiple potential long-term gradually evolving sequelae that are influenced by the type of injury, severity of the injury and medical interventions at the time of injury [8, 9]. Additionally, a link between mild traumatic brain injuries and Alzheimer’s disease or chronic traumatic encephalopathy has long been suspected [10]. At present, long-term effects of repeated TBI have been seen in multiple sports-related injuries, including post-traumatic parkinsonism, post-traumatic dementia and chronic post-concussion syndrome [11–14]. Thus, studying the short- and long-term consequences of TBI at a cellular and molecular level may lead to a new understanding and perhaps better long-term management of such injuries via new and/or refined treatment strategies.

Astrogliosis is a hallmark of TBI, which commences hours after injury and leads to an abnormal increase in the number of activated astrocytes in and around the injury site [15, 16]. Immediately after injury (acute phase), astrocytes are activated, becoming highly proliferative and up-regulating the production of extracellular proteins [17–19]. These astrocytes and their deposited extracellular matrix in and around the injury site form a glial scar. Over the years, a significant body of evidence has demonstrated that the glial scar contains molecules, such as chondroitin sulfate proteoglycans (CSPGs), that impede axonal growth, thus inhibiting neuronal regeneration [15, 20–23]. The intensity of the acute and chronic reactive astrogliosis, including the quantity and composition of the glial scar, affects immediate and long-term effects of TBI [6, 16, 24, 25]. Penetrating brain injuries (PBIs) cause direct parenchymal laceration, neuronal cell loss and hemorrhage, which lead to focal tissue damage at the injury site. Astrogliosis is triggered after TBIs forming a glial scar in and around the injury site [26–29]. Importantly, uninjured tissue bordering the injury site is also subject to astrogliosis, and the process of glial scarring therefore extends beyond the injury site [30]. Given the fact that glial scarring limits regeneration after injury, many studies have investigated whether limiting astrogliosis after injury, with particular focus on limiting deposition, could potentially promote regeneration [23, 27, 31–34]. Although many studies were able to demonstrate beneficial effects of limiting glial scarring on neuronal regeneration, many others were inconclusive or actually found there was an increased inflammatory response culminating in tissue damage beyond the injury site and an increase in neuronal loss. Thus, mounting evidence indicates that reactive astrocytes surrounding the injury site are instrumental in preserving the surrounding uninjured tissue by forming scar borders, which separate damaged and inflamed tissue from adjacent viable neural tissue [15, 16, 24, 35–40]. Sofroniew and colleagues elegantly demonstrated that targeting astrocytes after brain and spinal cord injury leads to increased inflammation, delayed recovery and increased neuronal loss [39, 41–44]. Moreover, the inhibition of astrocyte proliferation prolongs the healing period following central nervous system (CNS) injury [45]. Data from Hermann et al. show that GFAP-driven ablation of STAT3 in astrocytes leads to the loss of lesion demarcation and subsequent glial scar formation, and, in turn, results in increased invasion of inflammatory cells into adjacent viable tissue and further spread of inflammation [46]. This suggests that early glial scar formation by astrocytes restricts movement of inflammatory cells located within the lesion site into adjacent healthy tissue, thereby restricting tissue damage to the injury site. Thus, a recent body of evidence suggests scar tissue bordering the injury site is necessary for limiting inflammation and tissue damage to the injury site [37, 41]. There is currently a significant number of studies investigating how reactive astrocytes regulate and limit inflammation to the injury site, and which cellular components and major pathways could play a role in this process [20, 35, 41, 45, 47].

We recently found that tumor necrosis factor (TNF)-stimulated gene-6 (TSG-6) is secreted by astrocytes after injury and is a major constituent of the glial scar, but the role it plays within the glial scar remains to be established [48]. TSG-6 is a 35-kDa protein that is secreted by a wide range of cell types in response to inflammatory mediators and growth factors [49], and was originally identified as a gene product induced in fibroblasts by TNF [12]. TSG-6 contains a link module domain that mediates its interaction with the glycosaminoglycans (GAGs) hyaluronan (HA) and CS [49–51]. Our recent study identified that TSG-6 is expressed in the CNS, where it catalyzes the transfer of heavy chains (HCs) from Inter-a-Inhibitor (IαI, also known as ITI) onto HA, forming a specialized HA/HC/TSG-6 matrix within the glial scar, but the role of this specialized matrix within the glial scar remains to be established [48, 52–56]. This specific HA/HC/TSG-6 matrix has previously been shown to be monocyte-adhesive in other tissues and is believed to be present in most, if not all, inflammatory processes [57, 58]. These TSG-6 modified HA matrices bind inflammatory cells, and the interaction of these cells with this matrix modulates their responses, which are central to pathological inflammation [59–65]. The main objective of this study was to investigate the role TSG-6, a constituent of the glial scar, has in astrogliosis after a PBI. Given the well-characterized anti-inflammatory role of TSG-6 in other sites, the premise of this study was that TSG-6 could participate in the formation of an immunosuppressive environment within the glial scar. Our findings show that *TSG-6 null* mice present a more severe inflammatory response and increased glial scar deposition after injury when compared to littermate control mice. This increased inflammatory response in *TSG-6 null* mice was correlated with an enlarged area of astrogliosis beyond the injury site.

## Results

### TSG-6 expression after PBI

**Fig. 1:**
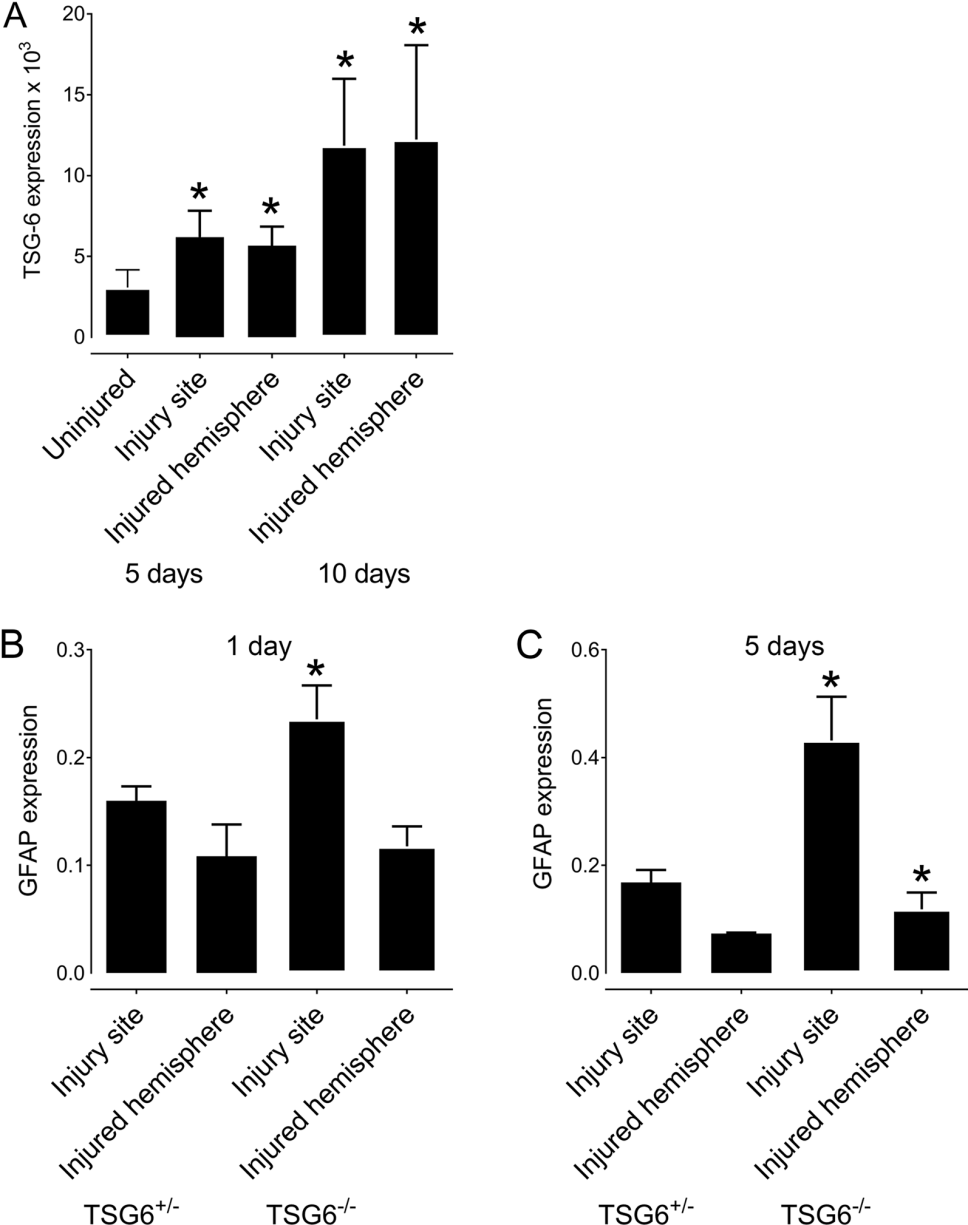
TSG-6 and GFAP expression after PBI. TSG-6 and GFAP mRNA expressions were quantified in the injury site and the injured hemisphere after PBI. **A** *TSG*^+/−^ mice were subjected to PBI, and the injury site and remaining injured hemisphere were collected 5 and 10 days after injury for analysis of TSG-6 expression. **B**, **C** *TSG*^+/−^ and *TSG-6*^−/−^ mice were subjected to PBI, and the injury site and remaining injured hemisphere were collected 1 day (**B**) and 5 days (**C**) after injury for analysis of GFAP expression. * = *p* ≤ 0.05 comparing *TSG-6*^−/+^ and *TSG-6*^−/−^ mice

In this study, transgenic *Tsg-6 null* mice (*Tnfip6*^*Δ/Δ*^), hereafter referred to as *Tsg-6*^−/−^ mice, and heterozygous mice, hereafter referred to as *Tsg-6*^+*/*−^ mice, were used to investigate the role of TSG-6 in the glial scar. In order to investigate whether TSG-6 is present in the glial scar after brain injury, we analyzed the expression profile of *Tsg6* in the injury site and injured hemisphere before and after a PBI in *Tsg-6*^+/−^ mice (Fig. 1A). There was a twofold increase in *Tsg-6* expression 5 days after injury when compared to uninjured mice. There was a further increase in *Tsg-6* expression over time after injury, with expression increasing two fold from 5 to 10 days after injury (Fig. 1A). Interestingly, we did not find a difference in the expression levels of *Tsg-6* between the injury site and the remaining hemisphere, indicating that *Tsg*-6 expression is not contained solely to the injury site (Fig. 1A). Therefore, there is also an increase in *Tsg-6* expression in the surrounding tissue after injury. No *Tsg-6* expression was identified in any of the samples from *Tsg-6*^−/−^ mice confirming that these mice are indeed *null* for *Tsg-6.*

### Analysis of astrocyte recruitment after PBI

We assessed the level of astrogliosis in the injury site and in the remaining injured hemisphere by quantifying the levels of GFAP^+^ astrocytes using real-time PCR (Fig. 1B, C). For such, we isolated mRNA from the injury site and remaining injured hemisphere 1 and 5 days after injury of *Tsg-6*^−/−^ and *Tsg-6*^+/−^ mice. Both *Tsg-6*^−/−^ and *Tsg-6*^+/−^ mice presented an increase in the levels of GFAP expression in the injury site when compared to the remaining injured hemisphere, which corroborates literature [36, 63, 64]. *Tsg-6*^−/−^ mice showed a significant increase in GFAP levels within the injury site at both 1 and 5 days post-injury when compared to *Tsg-6*^+/−^ mice (Fig. 1B, C). This data indicates that *Tsg-6*^−/−^ mice have more astrocytes in the injury site when compared to *Tsg-6*^+/−^ mice. At 5 days after injury, there was a significant increase in GFAP expression in the injured hemisphere of *Tsg-6*^−/−^ mice compared to *Tsg-6*^+/−^ mice, indicating that *Tsg-6*^−/−^ mice present astrogliosis beyond the injury site at 5 days post-injury.

### The effect of TSG-6 on the secretion of inflammatory markers after PBI

**Fig. 2:**
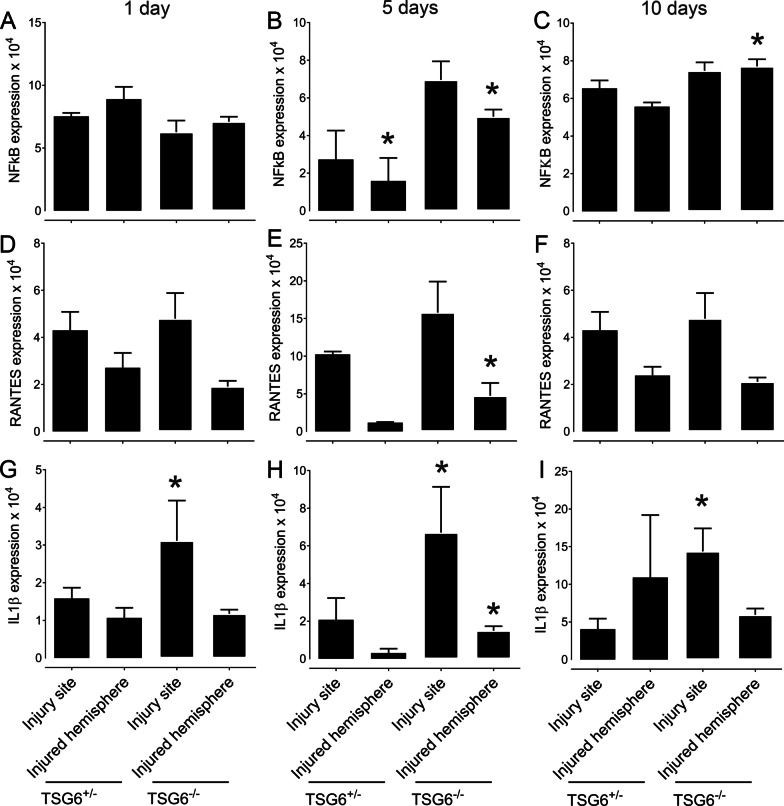
Analysis of inflammatory markers after PBI. NFκB, RANTES and IL1β mRNA expressions were quantified in the injury site and the injured hemisphere after PBI. *TSG*^+/−^ mice and *TSG-6*^−/−^ mice were subjected to PBI and the injury site and remaining injured hemisphere were collected 1, 5 and 10 days after injury. mRNA was extracted and subjected to real-time PCR analysis for NFκB (**A**–**C**), RANTES (**D**–**F**) and IL1β (**G**–**I**) mRNA expression. * = *p* ≤ 0.05 comparing *TSG-6*^−/+^ and *TSG-6*^−/−^ mice

The inflammatory response was also assessed in *Tsg-6*^−/−^ and *Tsg-6*^+/−^ mice 1, 5 and 10 days post-injury by quantifying the expression levels of *NFκB*, *Rantes* and *IL1β* (Fig. 2). Higher expression levels of *NFκB*, *Rantes* and *IL1β* were detected in *Tsg-6*^−/−^ mice when compared to *Tsg-6*^+/−^ mice during the acute phase after injury. Specifically, a ~ 2.5-fold and threefold increase in *NfκB* expression was found in the injury site and remaining injured hemisphere, respectively, in *Tsg-6*^−/−^ mice compared to *Tsg-6*^+/−^ mice 5 days after injury (Fig. 2B). 10 days after injury there was still a significant increase in *NfκB* expression in the surrounding hemisphere of *Tsg-6*^−/−^ mice when compared to *Tsg-6*^+/−^ mice (Fig. 2C). No significant differences were found in the expression of *NfκB* between *Tsg-6*^−/−^ and *Tsg-6*^+/−^ mice 1 day after injury (Fig. 1A). The levels of *Ccl5* (*Rantes)* were also assessed 1, 5 and 10 days post-injury. There was a significant increase in the expression of *Rantes* in the injured hemisphere of *Tsg-6*^−/−^ mice when compared to *Tsg-6*^+/−^ mice (a four fold increase) 5 days after injury; however, no difference was found between *Tsg-6*^−/−^ and *Tsg-6*^+/−^ mice 1 and 10 days post-injury (Fig. 2D–F). *IL1β* levels were increased in the injury site of *TSG-6*^−/−^ mice when compared to *Tsg-6*^+/−^ mice at 1 day post-injury (Fig. 2G). At 5 days post-injury a threefold and fourfold increase in the expression of *IL1β* were noted in the injury site and remaining injured hemisphere, respectively, of *Tsg-6*^−/−^ mice when compared to *Tsg-6*^+/−^ mice (Fig. 2H). At 10 days post-injury, a 2.5-fold increase in the expression of *IL1β* was noted in the injury site of *Tsg-6*^−/−^ mice when compared to *Tsg-6*^+/−^ mice (Fig. 2I).

### The effect of TSG-6 on the activation of microglia and infiltration of macrophages into the injury site after PBI

**Fig. 3:**
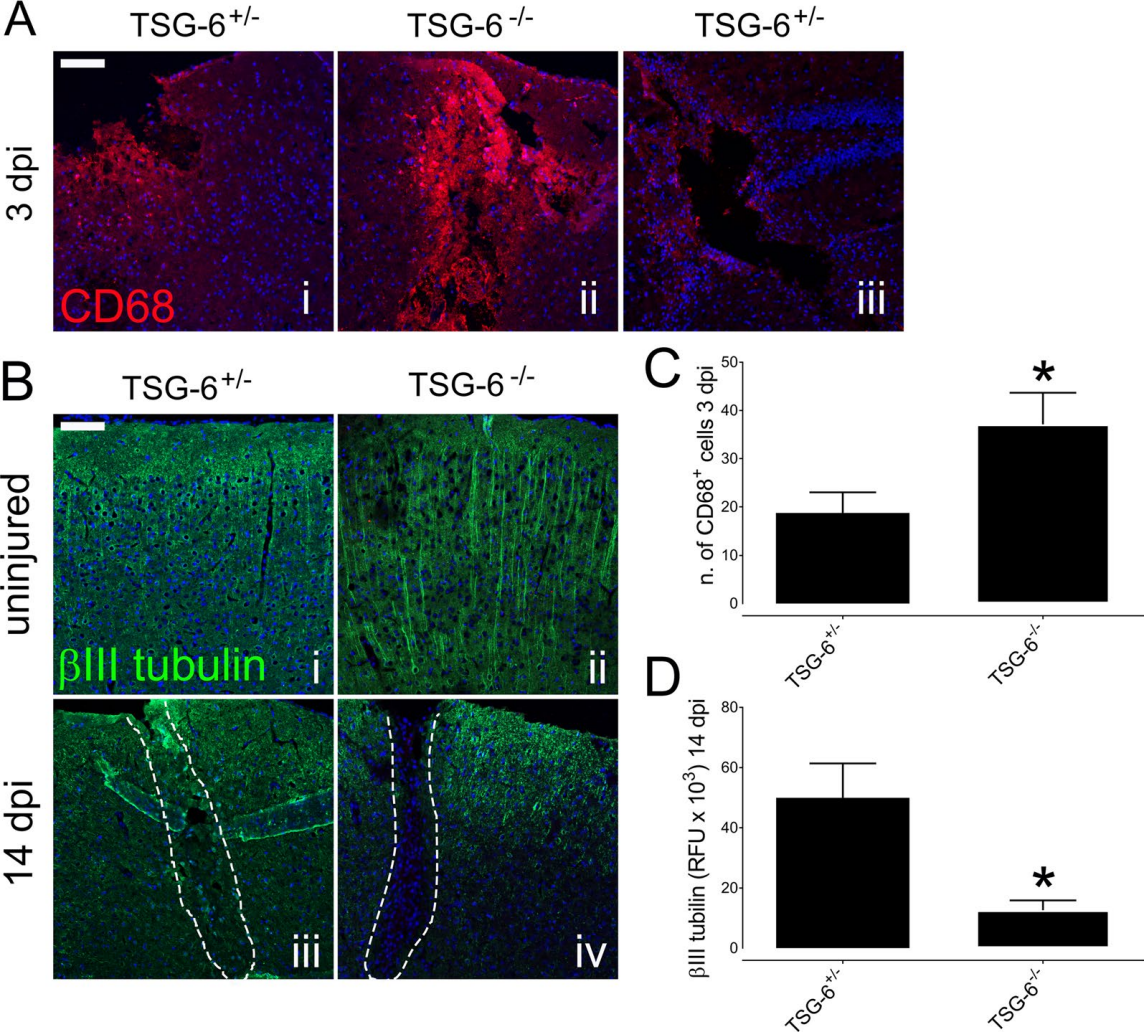
Analysis of inflammatory cell infiltration and neuronal cell loss after PBI. The distribution of macrophages and activated microglia was evaluated within the injury site of *TSG*^+/−^ and *TSG-6*^−/−^ mice 3 days post-injury (dpi) by anti-CD68 immunostaining (red) (**A**). Neuronal cells were immunostained with anti-β III tubulin (green) in the equivalent area of uninjured *TSG*^+/−^ (i) and *TSG-6*^−/−^ (ii) mice and within the injury site of *TSG-6*^−/+^ (iii) and *TSG-6*^−/−^ (iv) mice 14 days post-injury (dpi). The number of CD68^+^ cells was counted in the injury site and within 100 μm of the wound edge of *TSG-6*^−/+^ and *TSG-6*^−/−^ mice 3 days post-injury (**C**). The relative fluorescent units (RFU) of anti-β III tubulin staining were quantified in and around the injury site of *TSG-6*^−/+^ and *TSG-6*^−/−^ mice 14 days post-injury (**D**). Nuclei were counterstained with DAPI. Scale bar represents 100 μm. * = *p* ≤ 0.05 comparing *TSG-6*^−/+^ and *TSG-6*^−/−^ mice

In order to assess the inflammatory response in *Tsg-6*^−/−^ and *Tsg-6*^+/−^ mice, we also evaluated the number of CD68^+^ cells present within the injury site at 3 days post-injury (Fig. 3A, C). CD68 is routinely used as a marker for macrophages and activated microglia. There was a significant increase in the number of CD68^+^ cells in and around the injury site of *Tsg-6*^−/−^ mice when compared to *Tsg-6*^+/−^ mice (Fig. 3A panels i and ii). Importantly, even when analyzing deeper regions of the injury site of *Tsg-6*^+/−^ mice, the level of CD68^+^ cell infiltration was not as intense as that observed in *Tsg-6*^−/−^ mice (Fig. 3A panel iii). The combined number of CD68^+^ cells in the injury site and within a range of 100 μm from the wound edge was counted from images obtained from 2 different sections from at least 5 mice from each experimental point (Fig. 3C). A two fold increase in CD68^+^ cells was found in *Tsg-6*^−/−^ mice when compared to *Tsg-6*^+/−^ mice (Fig. 3C).

### Correlation between increased inflammatory response and neuronal damage

In order to verify whether the increased inflammatory response observed in *Tsg-6*^−/−^ mice correlates with neuronal loss, the distribution of neurons in and around the injury site was analyzed in *Tsg-6*^−/−^ and *Tsg-6*^+/−^ mice 14 days post-injury (Fig. 3B). For such, β III tubulin was used as a tissue-specific marker for identifying neurons within injured and non-injured brains. The distribution of β III tubulin can be seen in the equivalent region of uninjured *Tsg-6*^−/−^ and *Tsg-6*^+/−^ mice (Fig. 3 B panels i and ii). A significant increase in the area devoid of β III tubulin staining can be observed in and around the injury site of *Tsg-6*^−/−^ mice when compared to *Tsg-6*^+/−^ mice 14 days post-injury (Fig. 3B iii and iv). The relative fluorescence units (RLU) were quantified from an image of the injury site captured from at least 3 mice per experimental point. There was a fourfold decrease in β III tubulin staining in and around the injury site of *Tsg-6*^−/−^ mice when compared to *Tsg-6*^+/−^ mice 14 days post-injury (Fig. 3D).

### The effect of TSG-6 on the secretion of glial scar components after PBI

**Fig. 4:**
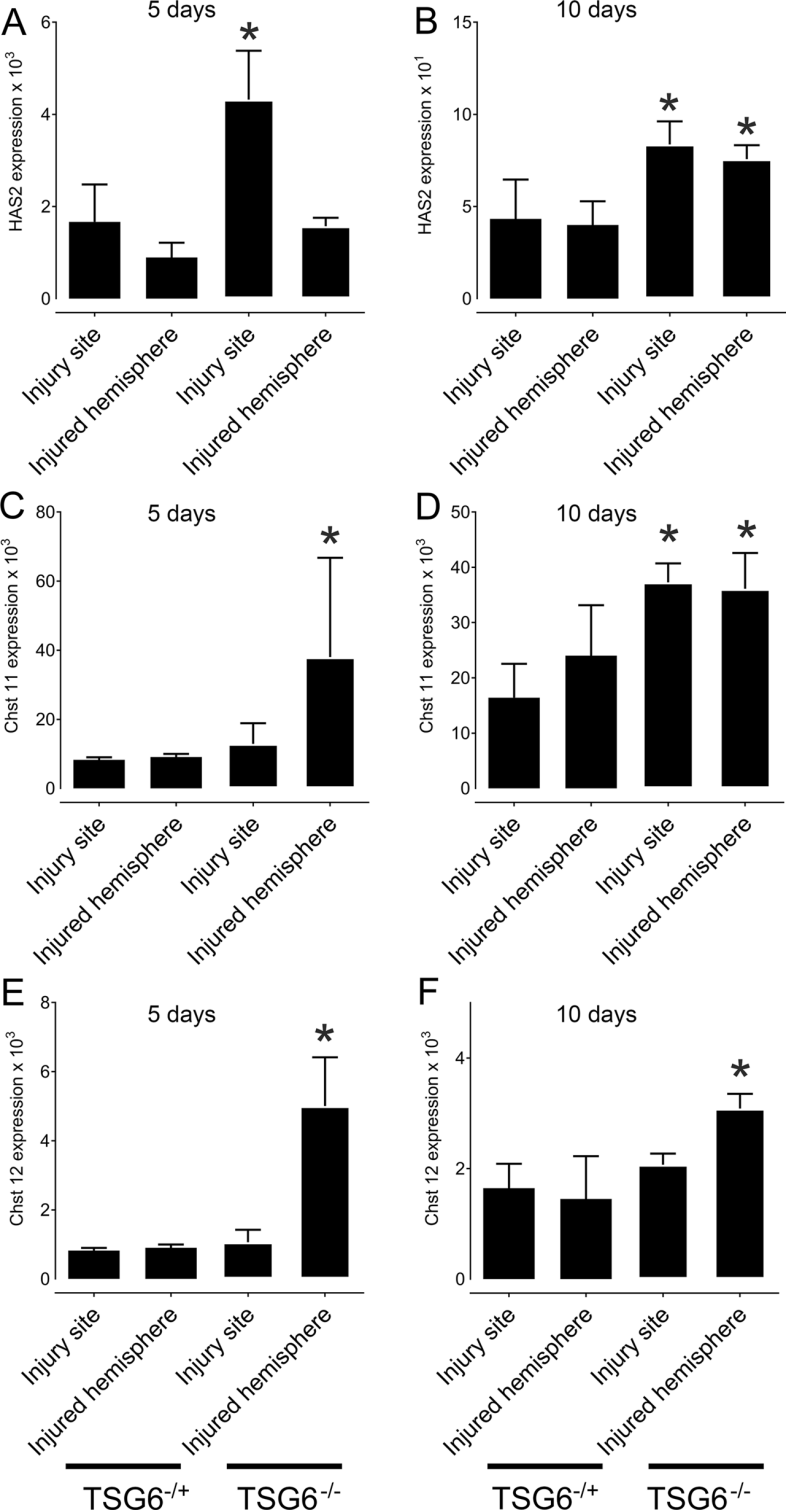
Analysis of glial scar extracellular matrix components after PBI. HAS2, Chst 11 and Chst 12 mRNA expression levels were quantified in the injury site and the injured hemisphere after PBI. *TSG*^+/−^ and *TSG-6*^−/−^ mice were subjected to PBI, and the injury site and remaining injured hemisphere were collected 5 and 10 days after injury. mRNA was extracted and subjected to real-time PCR analysis for HAS2 (**A**, **B**), Chst11 (**C**, **D**) and Chst 12 (**E**, **F**) mRNA expression. * = *p* ≤ 0.05 comparing *TSG-6*^−/+^ and *TSG-6*^−/−^ mice

We also evaluated glial scar secretion within the injury site and injured hemisphere by evaluating the expression levels of the biosynthetic enzymes responsible for HA and CS chain elongation, specifically hyaluronan synthase 2 (*Has2*), carbohydrate (chondroitin 4) sulfotransferase (*chst 11*) and carbohydrate (chondroitin 4) sulfotransferase 12 (*chst 12*) (Fig. 4). *Has2* expression increased in the injury site when compared to the remaining injured hemisphere 5 days post injury in both *Tsg-6*^+/−^ and *Tsg-6*^−/−^ mice, confirming the numerous previously published reports showing that HA is an integral component of the glial scar [17, 66–68]. Interestingly, there was a twofold increase in *Has2* expression in the injury site of *Tsg-6*^−/−^ mice when compared to *Tsg-6*^+/−^ mice 5 days after injury, indicating that there is a higher rate of glial scar production in *Tsg-6*^−/−^ mice when compared to *Tsg-6*^+/−^ mice (Fig. 4A). At 10 days post-injury, *Has2* expression was still increased by twofold in *Tsg-6*^−/−^ mice when compared to *Tsg-6*^+/−^ mice, but at this time point there was also an increase in *Has2* expression in the remaining injured hemisphere of *Tsg-6*^−/−^ mice when compared to *Tsg-6*^+/−^ mice (Fig. 4B). Thus, at 10 days after injury, in *Tsg-6*^−/−^ mice, the expression of glial scar components was no longer limited to the injury site, but was also present within the remaining injured hemisphere. Interestingly, this was also true for the expression of *Chst11* and *Chst12*, which showed a five fold and four fold increase, respectively, within the injured hemisphere of *Tsg-6*^−/−^ mice at 5 days post injury when compared to *Tsg-6*^+/−^ mice (Fig. 4C, E). The increase in *Chst11* and *Chst12*, in both the injury site and injured hemisphere, was maintained through to 10 days post-injury (Fig. 4D, F).

### The effect of TSG-6 on astrocyte activation and recruitment after PBI

**Fig. 5:**
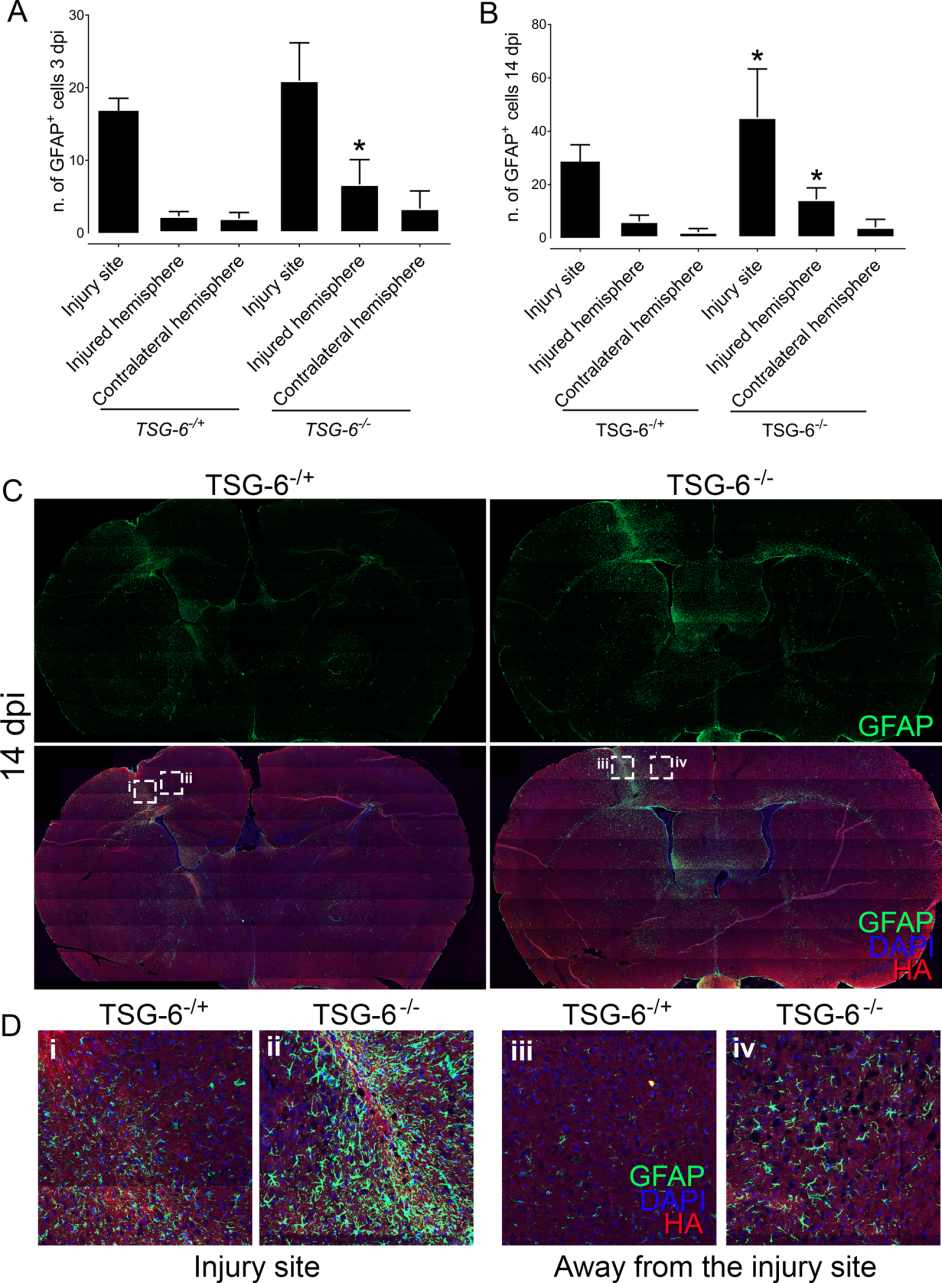
Analysis of astrocyte activation and recruitment after PBI. Brain sections from *TSG-6*^+/−^ and *TSG-6*^−/−^ mice were analyzed by immunofluorescence. Astrocytes were identified with anti-GFAP (green) and the glial scar with HABP (red). Nuclei were counterstained with DAPI (blue). Z-stacks were captured of the entire brain section using the tilling mode, and images were stitched together using Zen software. Thereafter, the number of astrocytes was counted within the injury site, within the injured hemisphere and in the contralateral hemisphere of brains 3 (**A**) and 14 dpi (**B**) in a double blinded manner. The distribution of astrocytes throughout the brain sections shows that in *TSG-6*^−/−^ mice the increase in astrocytes is not restricted to the injury site (**C**). Magnified images of the areas demarcated in (**C**) can be seen in (**D**). At least 3 mice were analyzed per genotype for each time point. * = *p* ≤ 0.05 comparing *TSG*^+/−^ and *TSG-6*^−/−^ mice

In order to further investigate the process of astrogliosis in *Tsg-6*^+/−^ and *Tsg-6*^−/−^ mice, injured brains were harvested and processed for histology. Sections were stained for GFAP in order to assess the distribution of astrocytes in and around the injury site, and, also, throughout the remaining brain tissue. The number of astrocytes (GFAP^+^ cells) was counted within the injury site, throughout the injured hemisphere, and, also, throughout the contralateral hemisphere 3 and 14 days post-injury (Fig. 5A, B). At 3 and 14 days post-injury there was a significant increase in the number of astrocytes within the injury site when compared to the injured hemisphere and contralateral hemisphere in both *Tsg-6*^+/−^ and *Tsg-6*^−/−^ mice. At 3 days post-injury there was no significant difference between the number of astrocytes within the injury site between *Tsg-6*^+/−^ and *Tsg-6*^−/−^ mice; however, there was a significant increase in the number of astrocytes within the injured hemisphere in *Tsg-6*^−/−^ mice when compared to *Tsg-6*^+/−^ mice (Fig. 5A). At 14 days post-injury there was a significant increase in the number of astrocytes within the injury site and injured hemisphere in *Tsg-6*^−/−^ mice when compared to *Tsg-6*^+/−^ mice (Fig. 5B, D). The increase in astrocytes can be seen beyond the injury site in *Tsg-6*^−/−^ mice (Fig. 5C, D panel iv).

## Discussion

Chondroitin sulfate proteoglycans (CSPGs) are well established as major extracellular matrix components in the central nervous system [69]. Over a decade ago, Silver et al. identified that CSPGs within the glial scar inhibit axonal growth, and this triggered a great deal of interest in targeting CS within the scar tissue as a means to promote axonal regeneration [32, 70–72]. Over the years, strategies utilizing the enzymes chondroitinase ABC (ChABC) and ChAC have been used to remove the CS component of the glial scar as a means to promote axonal growth and regeneration [50, 73–77]. Many studies have shown that specifically removing CS within the glial scar is enough for axons to grow across the injury site [32, 70, 78, 79]. However, significant regeneration was never observed in these studies, and many groups found limited or no improvement after targeting CS within the glial scar [70]. One unique characteristic of TSG-6 is its known ability to bind to a number of ligands including HA, CS and core proteins of proteoglycans (i.e. versican and aggrecan), forming specific HA/HC/TSG-6 and/or CS/HA/HC/TSG-6 matrices with immunosuppressive characteristics [61, 80–84]. Our previous study suggests these HA/HC/TSG-6 matrices are also present within the glial scar [48]. Therefore, given that TSG-6 directly binds to both HA and CS to form specific anti-inflammatory matrices, the ChABC and ChAC treatments used over the years to target the glial scar as a means to promote regeneration would also have removed TSG-6, a known anti-inflammatory molecule that is also a component of the glial scar [82]. The loss of TSG-6 by these treatments could, in part, explain why significant functional recovery was never obtained after ChABC and/or ChAC treatments.

To explore the role of TSG-6 in TBI, specifically in astrogliosis, we compared the differences in injury outcomes in *Tsg-6*^−/−^ and *Tsg-6*^+/−^ mice after PBIs. Our data show an increase in TSG-6 expression in the injured hemisphere of *Tsg-6*^+/−^ mice after TBI. This increase in expression of TSG-6 after CNS insults supports our earlier findings in a rat model that astrocytes secrete high levels of TSG-6 upon injury, which aids in the formation of a specialized HA/HC/TSG-6 matrix as part of an inflammatory response [48]. Since TSG-6 is known for having anti-inflammatory properties, to further study whether high levels of TSG-6 serve a purpose of rapidly suppressing inflammation after injury, we performed similar penetrating injuries in *Tsg-6*^−/−^ mice. We used immunofluorescence and RNA expression analyses of inflammatory and glial scar markers to elucidate the outcome during the acute phase and chronic phase of TBI. During the acute phase after injury, the observed increase in astrocyte activation, inflammatory cell infiltration and expression of inflammatory cytokines in *Tsg-6*^−/−^ mice indicate that the loss of TSG-6 results in a greater inflammatory response. Moreover, during the chronic phase of injury, unrestricted inflammatory response was observed throughout the injured hemisphere and was not limited to the injury site, as is seen after normal glial scar formation. Thus, injured *Tsg-6*^−/−^ mice appear to experience more severe tissue damage than their *Tsg-6*^+/−^ counterpart, both within and around the injury site. Thus, the loss of TSG-6 allows the damage to spread from the injury site to neighboring healthy tissues. We postulate that the cause of such widespread damage is due to the lack of the specialized HA-TSG6 or HA/HC/TSG-6 matrix, which could possibly serve to stabilize the glial scar and form an immunosuppressive environment, thereby protecting adjacent tissue from further damage. This hypothesis is further supported by the increase in CSPG and HA biosynthesis, both glial scar components, in *Tsg-6*^−/−^ mice. Specifically, these mice show increased *Has2*, *Chst11* and *Chst12* expression levels in tissues collected after the onset of glial scarring, and, also, during the chronic phase of astrogliosis, indicating an increase in scar tissue formation. This increase in expression was not only observed at the injury site, but also throughout the whole injured hemisphere, suggesting that the tissue damage spreads beyond the injury site in the absence of TSG-6. Collectively, these results demonstrate that the loss of TSG-6 leads to a more severe inflammatory response and, consequently, increased scarring after TBI. Thus, our results support the hypothesis put forward by many groups over the past decade that preventing the formation of the glial scar leads to inflammation and damage beyond the injury site. We also provide experimental evidence that shows that the glial scar functions to restrict the damage to the injury site. Importantly, these findings should be taken into account when attempts are made to disrupt the glial scar as a means to promote neuronal regeneration, since preventing formation of the glial scar may not have the beneficial outcomes as previously presumed.

## Conclusion

Our results show that TSG-6 has an anti-inflammatory role in the glial scar. Our study further supports the hypothesis that the glial scar forms a protective border surrounding the injury site thereby preventing the spread of inflammation and damage beyond the injury site.

## Methods

### TSG6 null (TSG6^−/−^) or heterozygous (TSG6^+/−^) mice and animal maintenance

Transgenic *Tsg-6 null* mice (*Tnfip6*^*Δ/Δ*^), herein referred to as *Tsg-6*^−/−^ mice, and heterozygous mice, herein referred to as *Tsg-6*^+/−^ mice, were maintained as previously described [56]. Our previously published work demonstrated that *Tsg-6*^+/−^ mice present a similar distribution of astrocytes throughout the brain to wild-type (wt) mice [48]. Moreover, *Tsg-6*^+/−^ mice have previously been shown not to display a phenotype and present similar TSG-6 expression levels as wt mice, and were therefore used as littermate controls in our study [56]. Experimental procedures for handling the mice were approved by the Institutional Animal Care and Use Committee (IACUC), University of Houston under protocol 16-036.

### Brain injury

Mice (7 to 8 weeks old) were anesthetized with ketamine (80–100 mg/kg—Vedco INC, Catalog# 07-890-8598) and xylazine (5–10 mg/kg, Akorn INC, Catalog# 07-808-1947) by IP injection and allowed to go into full anesthetic state. A sterile surgical drill (Precision Tools, Model Craft PPV2237) was used to make a hole of approximately 1.5 mm in diameter in the skull over the right frontal cortex at the stereotaxic coordinates AP: 1.0 mm, ML: 1.5 mm, and DV: 1.5 mm, according to Franklin and Paxinos (85). A 30-gauge needle (Exel, Catalog# 26437) was then used to make a puncture wound at a depth of 2 mm. After injury, the skin at the surgical site was closed with two sutures. The area was then cleaned with 70% ethanol, and mice were placed on a heating pad and monitored until they regained consciousness prior to being transferred to a clean cage. All surgeries were carried out at the same time of day to minimize bias. Mice were monitored daily and did not show any decrease in weight ≥ 15% when compared to their pre-surgical weight. Mice were euthanized, as outlined below, at 1, 3 and 5 days post injury to study the acute effects of brain injury, and at 10 and 14 days to study long-term/chronic effects. Five mice per experimental group were used for the real-time PCR analysis and at least seven mice per experimental group were used for immunofluorescence analysis.

### Perfusion fixation and brain tissue processing

Brain samples were collected at 1, 3, 5, 10, and 14 days post injury for immunofluorescence analyses. Briefly, mice were initially injected with a lethal dose of combined anesthetics containing 200 mg of ketamine and 40 mg xylazine. Final dosage received was 3 mg of ketamine and 0.6 mg of xylazine per mouse. Once mice were under deep anesthesia, abdominal and thoracic excisions were performed to expose the heart, which was used to perfuse 2% formalin (Fisher Scientific, Catalog# SF100-4) throughout the whole body via a gravity-driven flow system for whole body fixation. Subsequently, the brain was isolated from the skull and further immerse fixed for 2 days in 2% paraformaldehyde (Electron Microscopy Sciences, Catalog# 15710). For cryosection processing, brains were immersed in 30% sucrose for 2 days, embedded in OCT embedding medium (Fisher Healthcare, Catalog# 4585) and frozen. Sections 10 μm thick were obtained, mounted on superfrost slides (VWR, Catalog# 48311-703) and stored at − 20 °C until use.

### Immunofluorescence

Upon use, the slides were heated at 65 °C for 30 min and, subsequently, sections were washed with PBS to remove tissue freezing medium. Sections were then treated with 0.1% glycine (Fisher Chemical, Catalog# G46-500), blocked with 5% FBS (Seradigm, Catalog# 3100-500) and permeabilized with 0.1% saponin prepared in PBS. Sections were then incubated with the primary antibodies anti-Tenascin (Abcam, Ab108930), anti-GFAP (Abcam, Ab4647), anti-CD68 (Abcam, Ab31630) and anti-β III tubulin (Covance, PRB-435P-100). Sections were washed and incubated with appropriate secondary donkey antibodies conjugated with Alexa Fluor® 488 (Life Technologies) or Alexa Fluor® 555 (Life Technologies) for one hour at 18 °C. For HA staining, tissues were incubated with biotinylated HA binding protein (385911, Millipore) followed by NeutrAvidin®Alexa 555 (Life Technologies). The tissues were then washed and nuclei stained with 4’,6-diamidino-2-phenylindole (DAPI, Sigma-Aldrich). Sections were mounted in Prolong®Gold (Molecular Probes) and imaged using a ZEISS LSM 800 Confocal microscope with Airyscan. Secondary controls were done with a goat IgG isotype control (ab37388; Abcam) in place of the primary antibody and did not yield any significant staining (results not shown). For imaging, multiple z-stack tiles were captured of entire brain sections and frames were processed together into a single image (using the stitching mode followed by full orthogonal projection) using Zen Software (Zeiss). The number of GFAP^+^ and CD68^+^ cells in and around the injury site were counted by two independent investigators in a blinded manner and the relative Fluorescent intensity was measured using the Zen Software (Zeiss). At least 2 sections were scanned and analyzed from each animal for each set of antibodies and representative images shown in the figures.

### RNA extraction from brains and real-time PCR analysis

Brains collected from injured mice at 1, 5 and 10 days post injury were used for RNA extraction. At least 5 mice were used per experimental group and each animal was analyzed separately. Briefly, mice were euthanized and brain tissue was immediately isolated from each mouse. Injury sites (A samples) were dissected from the rest of the injured right hemisphere, transferred into a labeled Eppendorf tube and immediately immersed in liquid nitrogen. The remaining right hemisphere brain tissue (B samples) from each animal was transferred into a different tube and frozen as described. The samples were kept at − 80 °C until RNA extraction. Total RNA was isolated from these tissue samples using Trizol® Reagent (Invitrogen, Carlsbad, CA) and chloroform extraction (Sigma-Aldrich, Catalog# 650498). First strand cDNA was reverse transcribed using 1.5 to 2 μg of total RNA and the high capacity cDNA Reverse Transcription kit (Applied Biosystems, catalog# 4368814, lot 00593854), according to the manufacturer’s instructions. Quantitative real-time PCR amplification was performed on 1 μg or 50 ng of the cDNA (1:5) using the PowerUp SYBR Green Master Mix kit (Applied Biosystems, Catalog# A25918) in a CXF Connect Real-time System from BIO-RAD, using an activation cycle of 95 °C for 10 min, 40 cycles of 95 °C for 15 s and 60 °C for 1 min. A complete list of primers used in this study is shown in Table 1. Gene expression levels were normalized against *Actb* and *Gapdh* using the 2^−ΔCt^ and/or 2^−ΔΔCt^ methods.

**Table 1:** Primers used for real time PCR analysis

Gene (Mus musculus)	Accession number	Forward (5’ → 3’)	Reverse (5’ → 3’)
Tenascin C (tnc)	NM_011607.3	CCAGGGTTGCCACCTATTT	GTCTAGAGGATCCCACTCTACTT
Gfap	NM_001131020.1	AACAACCTGGCTGCGTATAG	TCTCGAACTTCCTCCTCATAGAT
Tsg-6 /Tnfaip6	NM_009398.2	CCCACATGCAAAGGAGTGTG	TGAGCCGAATGTGCCAGTAG
Chst1	NM_021439.2	CACCCAGTCATGCGGAGGAA	GCAGGATGGCAGTGTTGGAT
Chst12	NM_021528.3	GAGCTGGAGAACGAAGAGTTT	CAGGAGGTACTGGATGAAGTTG
IL1β	NM_008361.4	GTGCAAGTGTCTGAAGCAGC	CTCATCACTGTCAAAAGGTGGC
Cspg4	NM_139001.2	TCTACAGCTCCTGCCTCCTT	ATGTGGAGAACTGGAGCAGC
Ccl5 (Rantes)	NM_013653.3	CCTCACCATATGGCTCGGAC	ACGACTGCAAGATTGGAGCA
Nfkb1	NM_008689.2	GTCACCCATGGCACCATAAA	CCTTCACCTTCAGTTTCCTTCTC
Has1	NM_008215.2	CTA TGC TAC CAA GTA TAC CTC G	TCT CGG AAG TAA GAT TTG GAC
Has2	NM_008216.3	CGG TCG TCT CAA ATT CAT CTG	ACA ATG CAT CTT GTT CAG CTC
Has 3	NM_008217.4	GAT GTC CAA ATC CTC AAC AAG	CCC ACT AAT ACA TTG CAC AC
Itih1		CCA CCC CAT CGG TTT TGA AGT GTC T	TGC CAC GGG TCC TTG CTG TAG TCT
Itih2		ATG AAA AGA CTC ACG TGC TTT TTC	ATT TGC CTG GGG CCA GT
Itih3		TGA GGA GGT GGC CAA CCC ACT	CGC TTC TCC AGC AGC TGC TC
Actb	NM_007393.5	CACTGTCGAGTCGCGTCC	TCATCCATGGCGAACTGGTG
Gapdh	NM_001289726.1	AACAGCAACTCCCACTCTTC	CCTGTTGCTGTAGCCGTATT

### Statistical analysis

All values are presented as the mean ± standard deviation of the mean. The difference between the two groups was compared by means of the Student’s t-test. *p* ≤ 0.05 was considered to be statistically significant. Statistical analysis was performed using the GraphPad Prism version 7 software package (GraphPad Software, San Diego, CA, USA). * was used to indicate statistical differences of ≤ 0.05. Unless indicated otherwise, * indicates the statistical difference of *Tsg-6*^−/−^ mice compared to *Tsg-6*^+/−^ mice for each time point.

## Data Availability

The datasets used and/or analyzed during the current study are available from the corresponding author on reasonable request.

## Abbreviations

TBI: Traumatic brain injury
TNF: Tumor necrosis factor
TSG-6: TNF-stimulated gene-6
GFAP: Glial fibrillary acidic protein
VIM: Vimentin
CSPGs: Chondroitin sulfate proteoglycans
STAT3: Signal transducer and activator of Transcription 3
HA: Hyaluronan
GAGs: Glycosaminoglycans
CNS: Central nervous system
HC: Heavy chain
ITI or IαI: Inter-alpha inhibitor
hUMSCs: Human umbilical cord mesenchymal stem cells
PTX3: Pentraxin-3
NFKB: Nuclear factor kappa-light-chain-enhancer
IL1β: Interleukin 1 beta
Ccl5: Chemokine (C–C motif) ligand 1 or Rantes
HAS: Hyaluronan synthase
Chst: Carbohydrate (chondroitin 4) sulfotransferase
